# The genome sequence of the pear, *Pyrus communis* L.

**DOI:** 10.12688/wellcomeopenres.23426.1

**Published:** 2024-12-03

**Authors:** Markus Ruhsam, Royal Botanic Garden Edinburgh Genome Acquisition Lab, Darwin Tree of Life Barcoding collective, Plant Genome Sizing collective, Wellcome Sanger Institute Tree of Life Management, Samples and Laboratory team, Wellcome Sanger Institute Scientific Operations: Sequencing Operations, Wellcome Sanger Institute Tree of Life Core Informatics team, Tree of Life Core Informatics collective, Darwin Tree of Life Consortium

**Affiliations:** 1 Royal Botanic Garden Edinburgh, Edinburgh, Scotland, UK

**Keywords:** Pyrus communis, pear, genome sequence, chromosomal, Rosales

## Abstract

We present a genome assembly from a specimen of *Pyrus communis* (the pear; Streptophyta; Magnoliopsida; Rosales; Rosaceae). The genome sequence has a total length of 487.30 megabases. Most of the assembly is scaffolded into 17 chromosomal pseudomolecules. The mitochondrial and plastid genome assemblies have lengths of 443.53 kilobases and 159.93 kilobases, respectively. Gene annotation of this assembly on Ensembl identified 37,713 protein-coding genes.

## Species taxonomy

Eukaryota; Viridiplantae; Streptophyta; Streptophytina; Embryophyta; Tracheophyta; Euphyllophyta; Spermatophyta; Magnoliopsida; Mesangiospermae; eudicotyledons; Gunneridae; Pentapetalae; rosids; fabids; Rosales; Rosaceae; Amygdaloideae; Maleae; *Pyrus*; *Pyrus communis* L. (NCBI:txid23211).

## Background

*Pyrus communis* (the cultivated pear) is one of the most important temperate fruit crop trees ( Zohary *et al.*, 2012). The lack of discriminatory morphological characters and diagnostic genetic tools, however, have hampered the investigation of *Pyrus* diversification and domestication. Over the past 4000 years, pear cultivation has led to a huge number of cultivars through natural and artificial hybridisation ( Volk & Cornille, 2019). Armenia and the Caucasus are especially rich in wild pear forms.

Wild pears are self-incompatible trees but freely hybridise with the European domesticated crops where cultivars are interconnected by feral forms that often occur at edges of forests adjacent to cultivation ( Zohary *et al.*, 2012). The origin of the cultivated pear remains obscure but is thought to have arisen via hybridisation between the two eco-geographic wild subspecies *P. communis* ssp. *pyraster* (Europe) and *P. communis* ssp. *caucasica* (Asia) ( Zohary *et al.*, 2012). However, it is very likely that other wild pear species such as *P. spinosa*, *P. salicifolia*, *P. elaeagrifolia*, *P. syriaca* as well as some of the Eastern Asian species have contributed to the genetic variation of today’s domesticated pears ( Watkins, 1986; Zohary *et al.*, 2012).

*Pyrus communis* is generally a diploid species (2 *n* = 2 *x* = 34) although some triploid cultivars (2 *n* = 51) are known ( Yamamoto *et al.*, 2010; Zielinski & Thompson, 1967). This high-quality chromosome level genome of *P. communis* together with the draft genomes of the cultivar ‘Bartlett’ ( Chagné *et al.*, 2014; Li *et al.*, 2017; Linsmith *et al.*, 2019) may prove useful in elucidating the complex genetic origin of the cultivated pear. Here we present a chromosomal-level genome sequence for *Pyrus communis*, sequenced as part of the Darwin Tree of Life Project.

## Genome sequence report

**Figure 1.**
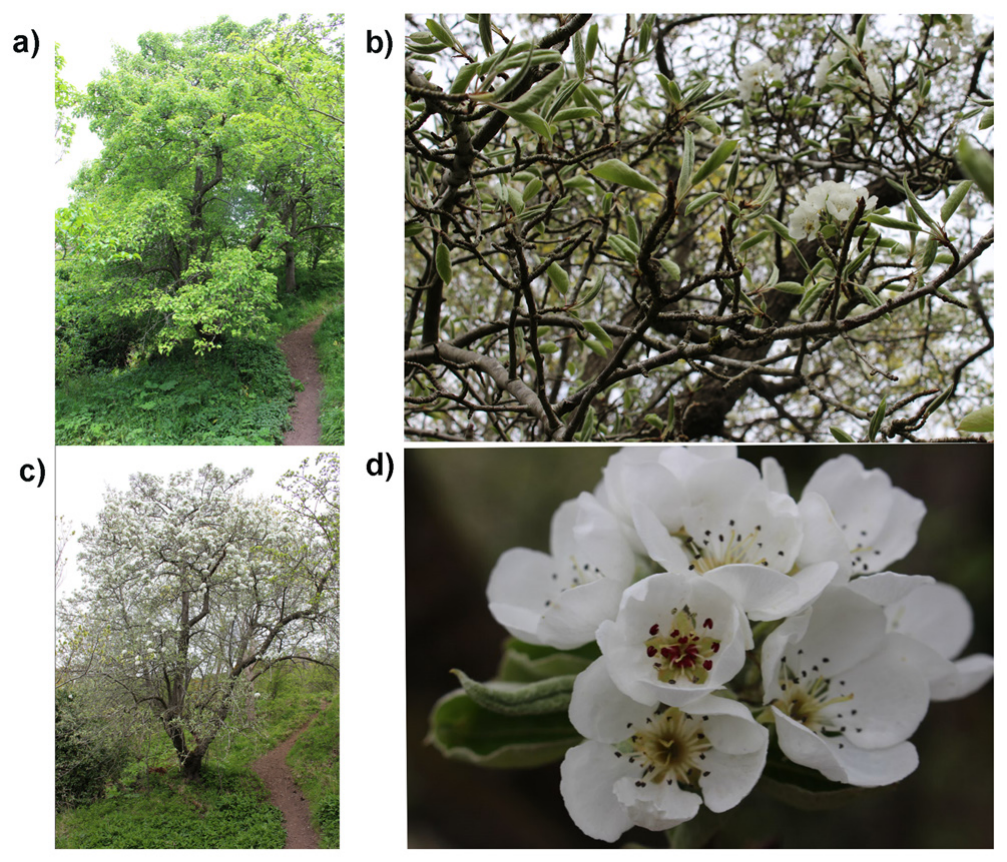
Photographs of the *Pyrus communis* (drPyrComm1) specimen from which samples were taken for genome sequencing.

The genome of a specimen of *Pyrus communis* ( Figure 1) was sequenced using Pacific Biosciences single-molecule HiFi long reads, generating a total of 27.95 Gb (gigabases) from 2.47 million reads, providing approximately 51-fold coverage. Using flow cytometry, the genome size (1C-value) was estimated to be 0.61 pg, equivalent to 590 Mb. Primary assembly contigs were scaffolded with chromosome conformation Hi-C data, which produced 116.42 Gb from 770.96 million reads, yielding an approximate coverage of 239-fold. Specimen and sequencing information is summarised in Table 1.

**Table 1.** Specimen and sequencing data for *Pyrus communis*.

Project information			
**Study title**	*Pyrus communis* (pear)		
**Umbrella BioProject**	PRJEB65225		
**Species**	*Pyrus communis*			
**BioSample**	SAMEA111431178		
**NCBI taxonomy ID**	23211		
Specimen information			
**Technology**	**ToLID**	**BioSample accession**	**Organism part**
**PacBio long read sequencing**	drPyrComm1	SAMEA111431238	leaf
**Hi-C sequencing**	drPyrComm1	SAMEA111431238	leaf
**RNA sequencing**	drPyrComm1	SAMEA111431238	leaf
Sequencing information			
**Platform**	**Run accession**	**Read count**	**Base count (Gb)**
**Hi-C Illumina NovaSeq 6000**	ERR11872592	7.71e+08	116.42
**PacBio Sequel IIe**	ERR11867222	2.47e+06	27.95
**RNA Illumina NovaSeq 6000**	ERR12245587	9.00e+07	13.59
**RNA Illumina NovaSeq 6000**	ERR12245588	7.16e+07	10.82

**Figure 2.**
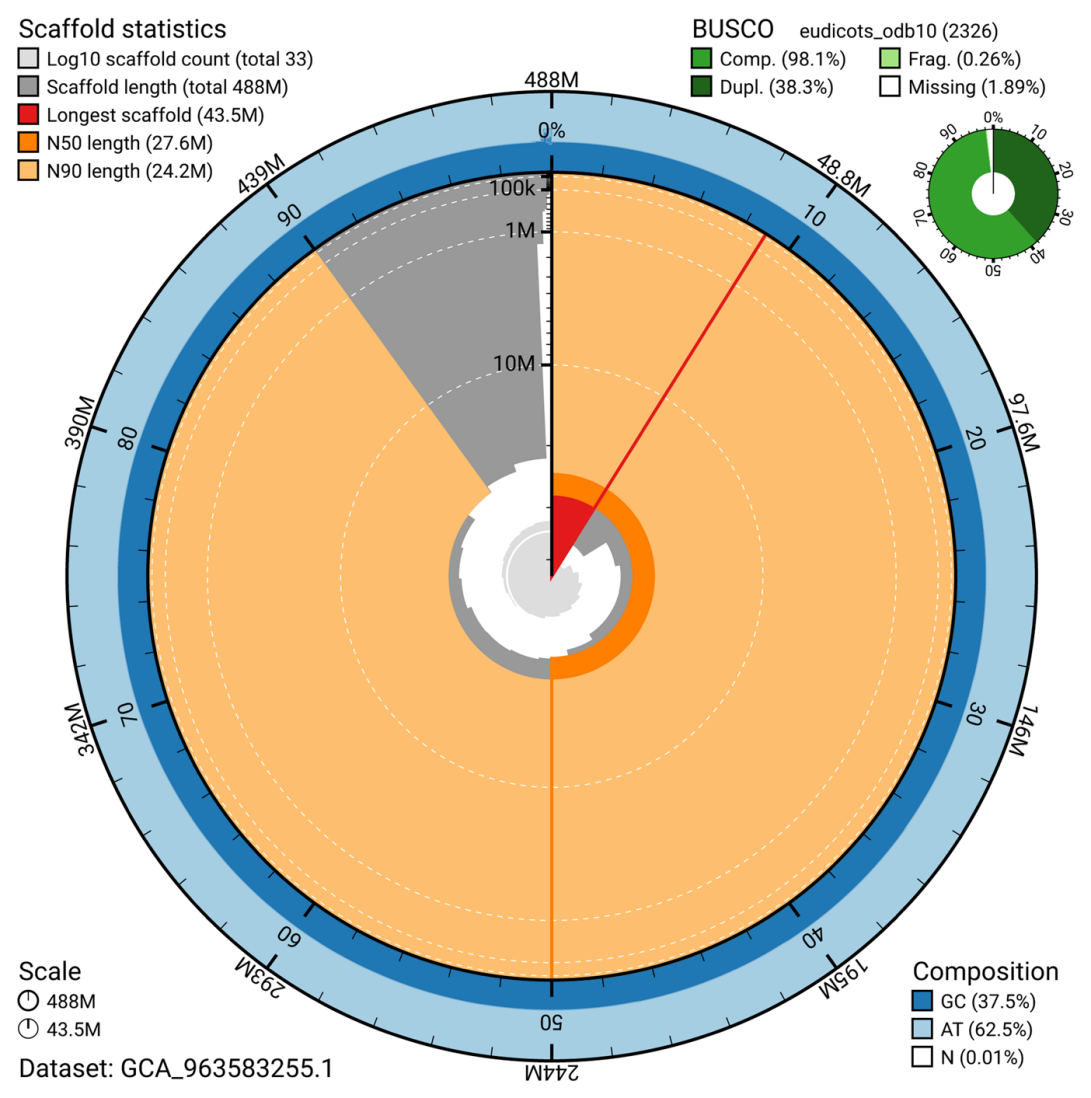
Snail plot summary of assembly statistics for assembly drPyrComm1.1: The BlobToolKit snail plot shows N50 metrics and BUSCO gene completeness. The main plot is divided into 1,000 size-ordered bins around the circumference with each bin representing 0.1% of the 487,940,690 bp assembly. The distribution of scaffold lengths is shown in dark grey with the plot radius scaled to the longest scaffold present in the assembly (43,463,786 bp, shown in red). Orange and pale-orange arcs show the N50 and N90 scaffold lengths (27,625,376 and 24,162,448 bp), respectively. The pale grey spiral shows the cumulative scaffold count on a log scale with white scale lines showing successive orders of magnitude. The blue and pale-blue area around the outside of the plot shows the distribution of GC, AT and N percentages in the same bins as the inner plot. A summary of complete, fragmented, duplicated and missing BUSCO genes in the eudicots_odb10 set is shown in the top right. An interactive version of this figure is available at https://blobtoolkit.genomehubs.org/view/GCA_963583255.1/dataset/GCA_963583255.1/snail.

**Figure 3.**
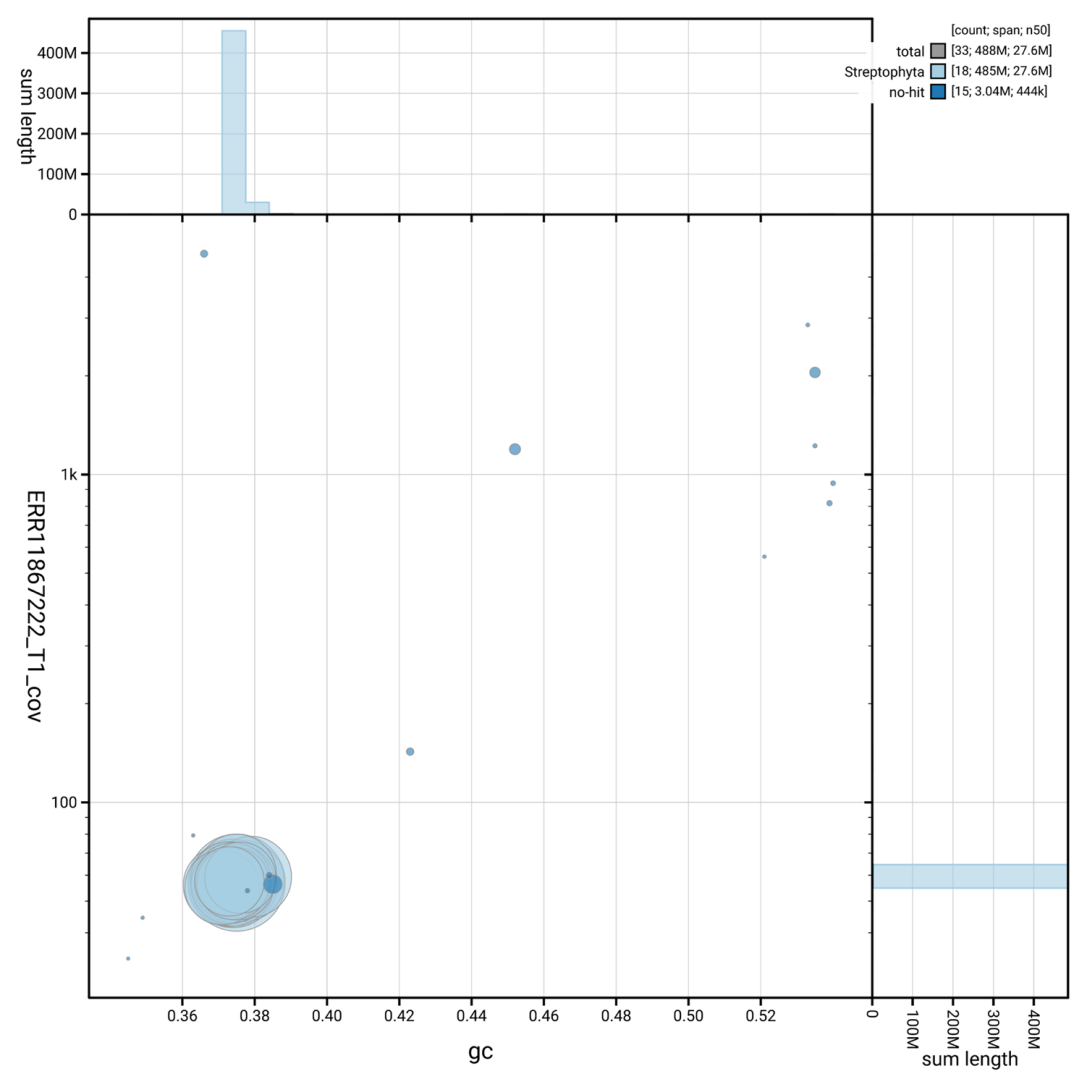
BlobToolKit blob plot for assembly drPyrComm1.1: BlobToolKit GC-coverage plot showing sequence coverage (vertical axis) and GC content (horizontal axis). The circles represent scaffolds, with the size proportional to scaffold length and the colour representing phylum membership. The histograms along the axes display the total length of sequences distributed across different levels of coverage and GC content. An interactive version of this figure is available at https://blobtoolkit.genomehubs.org/view/GCA_963583255.1/dataset/GCA_963583255.1/blob.

**Figure 4.**
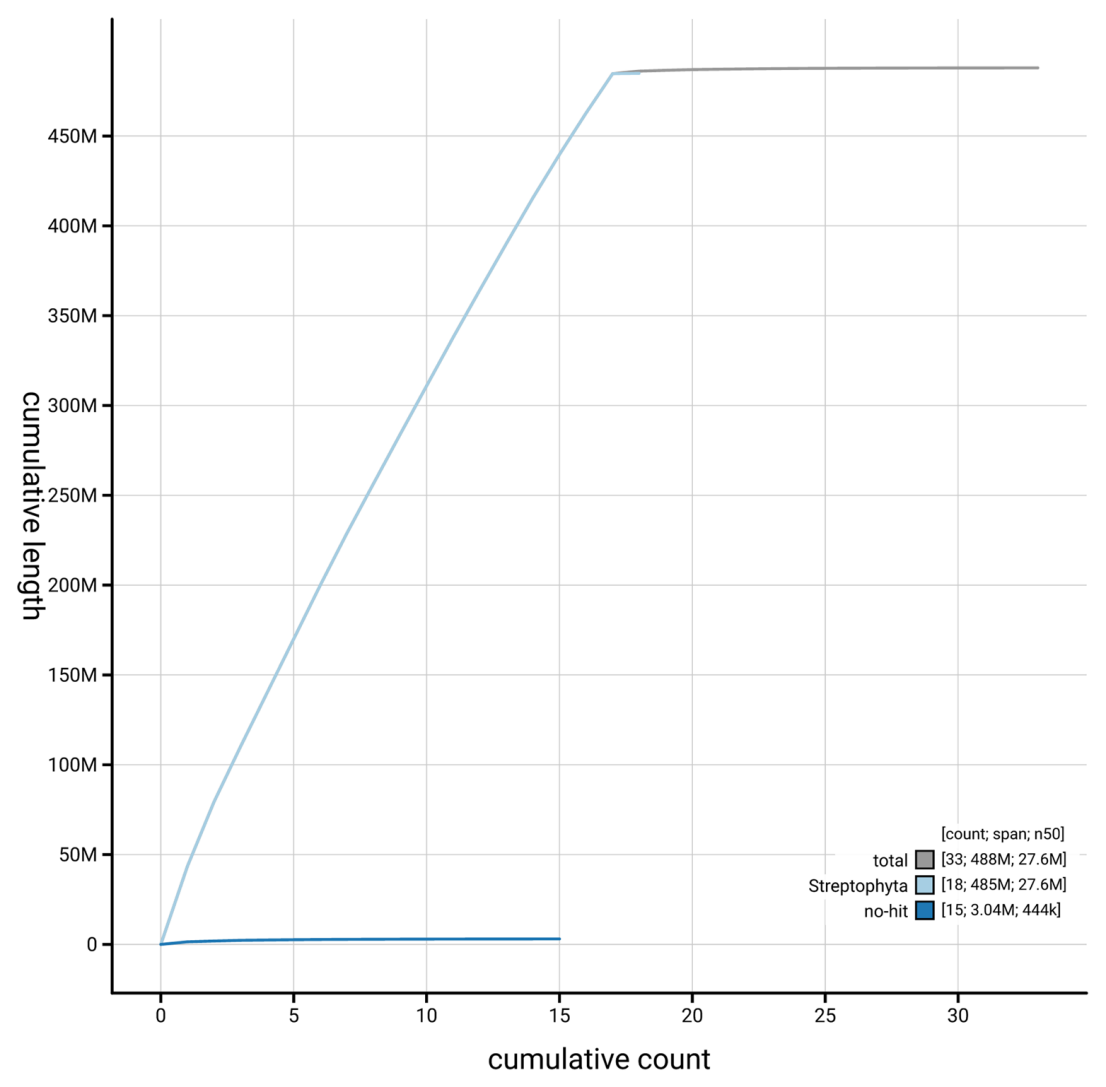
BlobToolKit cumulative sequence plot. The grey line shows cumulative length for all scaffolds. Coloured lines show cumulative lengths of scaffolds assigned to each phylum using the buscogenes taxrule. An interactive version of this figure is available at https://blobtoolkit.genomehubs.org/view/GCA_963583255.1/dataset/GCA_963583255.1/cumulative.

**Figure 5.**
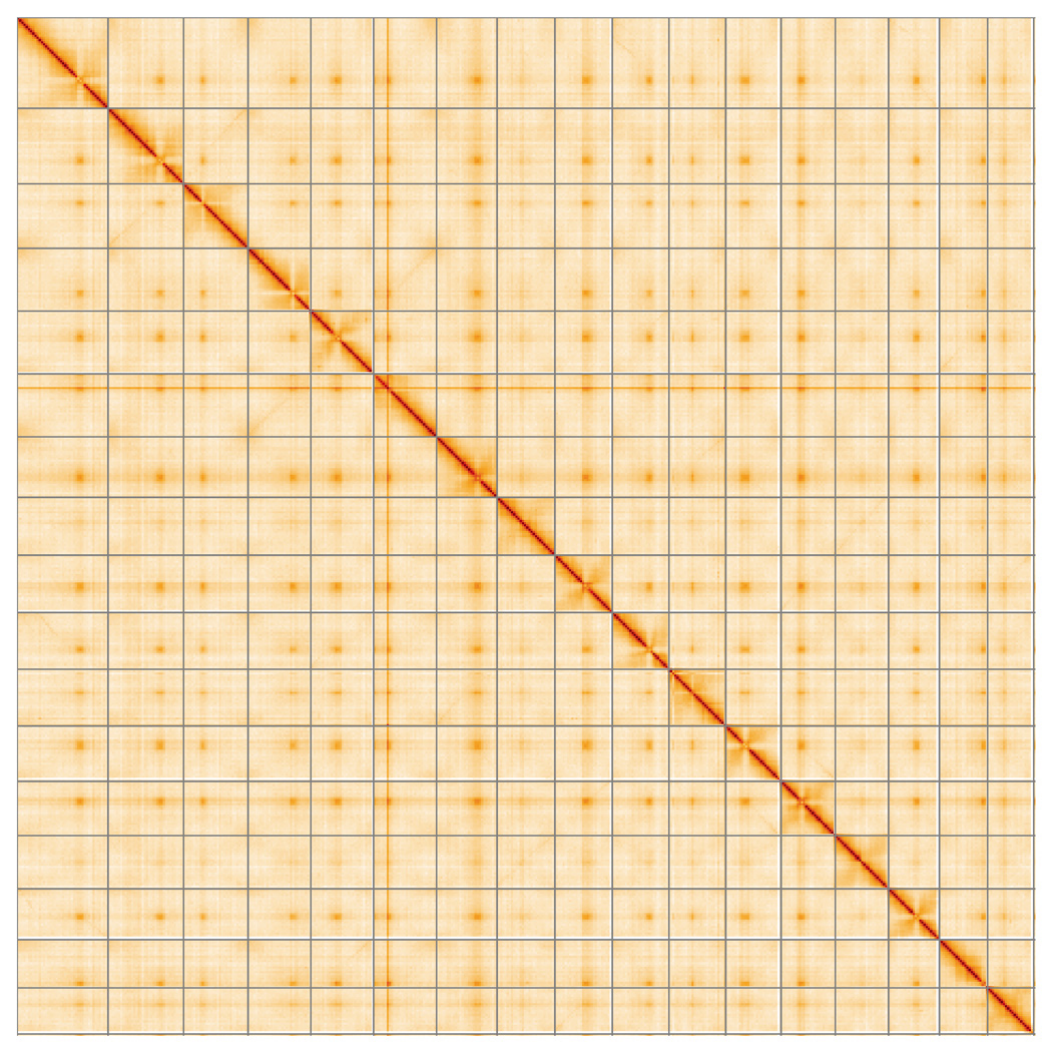
Genome assembly of *Pyrus communis*, drPyrComm1.1: Hi-C contact map of the drPyrComm1.1 assembly, visualised using HiGlass. Chromosomes are shown in order of size from left to right and top to bottom. Darker shades indicate more frequent physical contacts between regions, while lighter areas represent fewer contacts. An interactive version of this figure may be viewed at https://genome-note-higlass.tol.sanger.ac.uk/l/?d=WNUcOfo2RUaDG3C_PmW_CA.

Manual assembly curation corrected 32 missing joins or mis-joins and five haplotypic duplications, increasing the scaffold number by 3.12% and the scaffold N50 by 1.03%. The final assembly has a total length of 487.30 Mb in 31 sequence scaffolds, with 145 gaps. The scaffold N50 is 27.6 Mb ( Table 2). The snail plot in Figure 2 summarises the assembly statistics, while the blob plot in Figure 3 shows the distribution of assembly scaffolds by GC proportion and coverage. The cumulative assembly plot in Figure 4 shows curves for subsets of scaffolds assigned to different phyla. Most (99.33%) of the assembly sequence was assigned to 17 chromosomal-level scaffolds. Chromosome-scale scaffolds confirmed by the Hi-C data are named in order of size ( Figure 5; Table 3). While not fully phased, the assembly deposited is of one haplotype. Contigs corresponding to the second haplotype have also been deposited. The mitochondrial and plastid genomes were also assembled and can be found as contigs within the multifasta file of the genome submission.

**Table 2.** Genome assembly data for *Pyrus communis*, drPyrComm1.1.

Genome assembly		
Assembly name	drPyrComm1.1	
Assembly accession	GCA_963583255.1	
*Accession of alternate haplotype*	*GCA_963583335.1*	
Span (Mb)	487.30	
Number of contigs	178	
Contig N50 length (Mb)	4.8	
Number of scaffolds	31	
Scaffold N50 length (Mb)	27.6	
Longest scaffold (Mb)	43.46	
Assembly metrics		Benchmark*
Contig N50 (Mb)	4.8	*> 1 Mb*
Scaffold N50 (Mb)	27.6	*> 10 Mb*
Gaps/Gb	145 gaps total	*< 200*
Consensus quality (QV)	64.2	*> 50*
*k*-mer completeness	100.0%	*> 95%*
BUSCO **	C:98.1%[S:59.7%,D:38.4%], F:0.3%,M:1.6%,n:2,326	*C > 95%*
Percentage of assembly mapped to chromosomes	99.33%	*> 90%*
Organelles	Mitochondrial genome: 443.53 kb Plastid genome: 159.93 kb	*single complete allele*
Genome annotation of assembly GCA_963583255.1 at Ensembl		
Number of protein-coding genes	37,713	
Number of non-coding genes	9,611	
Number of gene transcripts	69,776	

Assembly metric benchmarks are adapted from column VGP-2020 (7.C.Q50) of “Table 1: Proposed standards and metrics for defining genome assembly quality” from Rhie *et al.* (2021). ** BUSCO scores based on the eudicotyledons_odb10 BUSCO set using version 5.4.3. C = complete [S = single copy, D = duplicated], F = fragmented, M = missing, n = number of orthologues in comparison. A full set of BUSCO scores is available at https://blobtoolkit.genomehubs.org/view/Pyrus_communis/dataset/GCA_963583255.1/busco.

**Table 3.** Chromosomal pseudomolecules in the genome assembly of *Pyrus communis*, drPyrComm1.

INSDC accession	Name	Length (Mb)	GC%
OY757068.1	1	43.46	37.5
OY757069.1	2	35.84	37.5
OY757070.1	3	30.84	37.5
OY757071.1	4	29.97	37.5
OY757072.1	5	29.93	37.5
OY757073.1	6	29.92	38.0
OY757074.1	7	28.87	37.5
OY757075.1	8	27.63	37.0
OY757076.1	9	27.34	37.5
OY757077.1	10	27.1	37.5
OY757078.1	11	27.01	37.5
OY757079.1	12	26.29	37.5
OY757080.1	13	25.89	37.5
OY757081.1	14	25.47	37.0
OY757082.1	15	24.16	37.5
OY757083.1	16	23.07	37.5
OY757084.1	17	21.92	37.5
OY757085.1	MT	0.44	45.0

The estimated Quality Value (QV) of the final assembly is 64.2 with *k*-mer completeness of 100.0%, and the assembly has a BUSCO v5.4.3 completeness of 98.1% (single = 59.7%, duplicated = 38.4%), using the eudicotyledons_odb10 reference set ( *n* = 2,326).

Metadata for specimens, BOLD barcode results, spectra estimates, sequencing runs, contaminants and pre-curation assembly statistics are given at https://links.tol.sanger.ac.uk/species/23211.

## Genome annotation report

The *Pyrus communis* genome assembly (GCA_963583255.1) was annotated at the European Bioinformatics Institute (EBI) on Ensembl Rapid Release. The resulting annotation includes 69,776 transcribed mRNAs from 37,713 protein-coding and 9,611 non-coding genes ( Table 2; https://rapid.ensembl.org/Pyrus_communis_GCA_963583255.1/Info/Index). The average transcript length is 3,179.76. There are 1.47 coding transcripts per gene and 5.25 exons per transcript.

## Methods

### Sample acquisition, DNA barcoding and genome size estimation

Leaf material of *Pyrus communis* (specimen ID EDTOL03996, ToLID drPyrComm1) was collected from Edinburgh, Scotland, United Kingdom (latitude 55.92, longitude –3.19) on 2022-05-31. The specimen was collected and identified by Markus Ruhsam (Royal Botanic Garden Edinburgh) and preserved by liquid nitrogen. The herbarium voucher associated with the sequenced plant is https://data.rbge.org.uk/herb/E01152516, deposited in the herbarium of RBG Edinburgh (E).

The initial species identification was verified by an additional DNA barcoding process following the framework developed by Twyford *et al*. (2024). Part of the plant specimen was preserved in silica gel desiccant ( Chase & Hills, 1991). DNA was extracted from the dried specimen, then PCR was used to amplify standard barcode regions. The resulting amplicons were sequenced and compared to public sequence databases including GenBank and the Barcode of Life Database (BOLD). The barcode sequences for this specimen are available on BOLD ( Ratnasingham & Hebert, 2007). Following whole genome sequence generation, DNA barcodes were also used alongside the initial barcoding data for sample tracking through the genome production pipeline at the Wellcome Sanger Institute ( Twyford *et al.*, 2024). The standard operating procedures for the Darwin Tree of Life barcoding have been deposited on protocols.io ( Beasley *et al.*, 2023).

The genome size was estimated by flow cytometry using the fluorochrome propidium iodide and following the ‘one-step’ method as outlined in Pellicer *et al.* (2021). For this species, the General Purpose Buffer (GPB) supplemented with 3% PVP and 0.08% (v/v) beta-mercaptoethanol was used for isolation of nuclei ( Loureiro *et al.*, 2007), and the internal calibration standard was *Solanum lycopersicum* ‘Stupiké polní rané’ with an assumed 1C-value of 968 Mb ( Doležel *et al.*, 2007).

### Nucleic acid extraction

The workflow for high molecular weight (HMW) DNA extraction at the WSI Tree of Life Core Laboratory includes a sequence of core procedures: sample preparation and homogenisation, DNA extraction, fragmentation and purification. Detailed protocols are available on protocols.io ( Denton *et al.*, 2023).

The drPyrComm1 sample of leaf tissue was weighed and dissected on dry ice ( Jay *et al.*, 2023), and cryogenically disrupted using the Covaris cryoPREP ^®^ Automated Dry Pulverizer ( Narváez-Gómez *et al.*, 2023). HMW DNA was extracted in the WSI Scientific Operations core using the Automated Plant MagAttract v2 protocol ( Todorovic *et al.*, 2023). HMW DNA was sheared into an average fragment size of 12–20 kb in a Megaruptor 3 system ( Bates *et al.*, 2023). Sheared DNA was purified by solid-phase reversible immobilisation, using AMPure PB beads to eliminate shorter fragments and concentrate the DNA ( Strickland *et al.*, 2023). The concentration of the sheared and purified DNA was assessed using a Nanodrop spectrophotometer and Qubit Fluorometer and Qubit dsDNA High Sensitivity Assay kit. Fragment size distribution was evaluated by running the sample on the FemtoPulse system.

RNA was extracted from leaf tissue of drPyrComm1 in the Tree of Life Laboratory at the WSI using the RNA Extraction: Automated MagMax™ *mir*Vana protocol ( do Amaral *et al.*, 2023). The RNA concentration was assessed using a Nanodrop spectrophotometer and a Qubit Fluorometer using the Qubit RNA Broad-Range Assay kit. Analysis of the integrity of the RNA was done using the Agilent RNA 6000 Pico Kit and Eukaryotic Total RNA assay.

### Hi-C preparation

Hi-C data were generated from leaf tissue of the drPyrComm1 sample at the WSI Scientific Operations core, using the Arima-HiC v2 kit. Tissue was finely ground using cryoPREP, and then subjected to nuclei isolation using a modified protocol of the Qiagen QProteome Kit. After isolation, the nuclei were fixed, and the DNA crosslinked using a 37% formaldehyde solution. The crosslinked DNA was then digested using the restriction enzyme master mix. The 5’-overhangs were then filled in and labelled with biotinylated nucleotides and proximally ligated. An overnight incubation was carried out for enzymes to digest remaining proteins and for crosslinks to reverse. A clean up was performed with SPRIselect beads prior to library preparation. DNA concentration was quantified using the Qubit Fluorometer v2.0 and Qubit HS Assay Kit according to the manufacturer’s instructions.

### Library preparation and sequencing

Library preparation and sequencing were performed at the WSI Scientific Operations core. Libraries were prepared using the PacBio Express Template Preparation Kit v2.0 (Pacific Biosciences, California, USA) as per the manufacturer’s instructions. The kit includes the reagents required for removal of single-strand overhangs, DNA damage repair, end repair/A-tailing, adapter ligation, and nuclease treatment. Library preparation also included a library purification step using AMPure PB beads (Pacific Biosciences, California, USA) and size selection step to remove templates <3kb using AMPure PB modified SPRI. DNA concentration was quantified using the Qubit Fluorometer v2.0 and Qubit HS Assay Kit and the final library fragment size analysis was carried out using the Agilent Femto Pulse Automated Pulsed Field CE Instrument and gDNA 165kb gDNA and 55kb BAC analysis kit. Samples were sequenced using the Sequel IIe system (Pacific Biosciences, California, USA). The concentration of the library loaded onto the Sequel IIe was between 40 - 135 pM. The SMRT link software, a PacBio web-based end-to-end workflow manager, was used to set-up and monitor the run, as well as perform primary and secondary analysis of the data upon completion.

For Hi-C library preparation, DNA was fragmented to a size of 400 to 600 bp using a Covaris E220 sonicator. The DNA was then enriched, barcoded, and amplified using the NEBNext Ultra II DNA Library Prep Kit, following manufacturers’ instructions. The Hi-C sequencing was performed using paired-end sequencing with a read length of 150 bp on an Illumina NovaSeq 6000.

Poly(A) RNA-Seq libraries were constructed using the NEB Ultra II RNA Library Prep kit, following manufacturer’s instructions, and RNA sequencing was performed on the Illumina NovaSeq 6000 instrument.

### Genome assembly, curation and evaluation

***Assembly***

The HiFi reads were first assembled using Hifiasm ( Cheng *et al.*, 2021) with the --primary option. Haplotypic duplications were identified and removed using purge_dups ( Guan *et al.*, 2020). The Hi-C reads were mapped to the primary contigs using bwa-mem2 ( Vasimuddin *et al.*, 2019). The contigs were further scaffolded using the provided Hi-C data ( Rao *et al.*, 2014) in YaHS ( Zhou *et al.*, 2023) using the --break option. The scaffolded assemblies were evaluated using Gfastats ( Formenti *et al.*, 2022), BUSCO ( Manni *et al.*, 2021) and MERQURY.FK ( Rhie *et al.*, 2020). The organelle genomes were assembled using OATK ( Zhou, 2023).

***Curation***

The assembly was decontaminated using the Assembly Screen for Cobionts and Contaminants (ASCC) pipeline (article in preparation). Flat files and maps used in curation were generated in TreeVal ( Pointon *et al.*, 2023). Manual curation was primarily conducted using PretextView ( Harry, 2022), with additional insights provided by JBrowse2 ( Diesh *et al.*, 2023) and HiGlass ( Kerpedjiev *et al.*, 2018). Scaffolds were visually inspected and corrected as described by Howe *et al*. (2021). Any identified contamination, missed joins, and mis-joins were corrected, and duplicate sequences were tagged and removed. The process is documented at https://gitlab.com/wtsi-grit/rapid-curation (article in preparation).

***Evaluation of final assembly***

The final assembly was post-processed and evaluated using the three Nextflow ( Di Tommaso *et al.*, 2017) DSL2 pipelines: sanger-tol/readmapping ( Surana *et al.*, 2023a), sanger-tol/genomenote ( Surana *et al.*, 2023b), and sanger-tol/blobtoolkit ( Muffato *et al.*, 2024). The readmapping pipeline aligns the Hi-C reads using bwa-mem2 ( Vasimuddin *et al.*, 2019) and combines the alignment files with SAMtools ( Danecek *et al.*, 2021). The genomenote pipeline converts the Hi-C alignments into a contact map using BEDTools ( Quinlan & Hall, 2010) and the Cooler tool suite ( Abdennur & Mirny, 2020). The contact map is visualised in HiGlass ( Kerpedjiev *et al.*, 2018). This pipeline also computes *k*-mer completeness and QV consensus quality values with FastK and MERQURY.FK, and runs BUSCO ( Manni *et al.*, 2021) to assess completeness.

The blobtoolkit pipeline is a Nextflow port of the previous Snakemake Blobtoolkit pipeline ( Challis *et al.*, 2020). It aligns the PacBio reads in SAMtools and minimap2 ( Li, 2018) and generates coverage tracks for regions of fixed size. In parallel, it queries the GoaT database ( Challis *et al.*, 2023) to identify all matching BUSCO lineages to run BUSCO ( Manni *et al.*, 2021). For the three domain-level BUSCO lineages, the pipeline aligns the BUSCO genes to the UniProt Reference Proteomes database ( Bateman *et al.*, 2023) with DIAMOND ( Buchfink *et al.*, 2021) blastp. The genome is also split into chunks according to the density of the BUSCO genes from the closest taxonomic lineage, and each chunk is aligned to the UniProt Reference Proteomes database with DIAMOND blastx. Genome sequences without a hit are chunked with seqtk and aligned to the NT database with blastn ( Altschul *et al.*, 1990). The blobtools suite combines all these outputs into a blobdir for visualisation.

The genome evaluation pipelines were developed using nf-core tooling ( Ewels *et al.*, 2020) and MultiQC ( Ewels *et al.*, 2016), relying on the Conda package manager, the Bioconda initiative ( Grüning *et al.*, 2018), the Biocontainers infrastructure ( da Veiga Leprevost *et al.*, 2017), as well as the Docker ( Merkel, 2014) and Singularity ( Kurtzer *et al.*, 2017) containerisation solutions.

Table 4 contains a list of relevant software tool versions and sources.

**Table 4.** Software tools: versions and sources.

Software tool	Version	Source
BEDTools	2.30.0	https://github.com/arq5x/bedtools2
Blast	2.14.0	ftp://ftp.ncbi.nlm.nih.gov/blast/executables/blast+/
BlobToolKit	4.3.7	https://github.com/blobtoolkit/blobtoolkit
BUSCO	5.4.3 and 5.5.0	https://gitlab.com/ezlab/busco
bwa-mem2	2.2.1	https://github.com/bwa-mem2/bwa-mem2
Cooler	0.8.11	https://github.com/open2c/cooler
DIAMOND	2.1.8	https://github.com/bbuchfink/diamond
fasta_windows	0.2.4	https://github.com/tolkit/fasta_windows
FastK	427104ea91c78c3b8b8b49f1a7d6bbeaa869ba1c	https://github.com/thegenemyers/FASTK
Gfastats	1.3.6	https://github.com/vgl-hub/gfastats
GoaT CLI	0.2.5	https://github.com/genomehubs/goat-cli
Hifiasm	0.16.1-r375	https://github.com/chhylp123/hifiasm
HiGlass	44086069ee7d4d3f6f3f0012569789ec138f42b84aa44357826c0b6753eb28de	https://github.com/higlass/higlass
Merqury.FK	d00d98157618f4e8d1a9190026b19b471055b22e	https://github.com/thegenemyers/MERQURY.FK
MultiQC	1.14, 1.17, and 1.18	https://github.com/MultiQC/MultiQC
NCBI Datasets	15.12.0	https://github.com/ncbi/datasets
Nextflow	23.04.0-5857	https://github.com/nextflow-io/nextflow
PretextView	0.2	https://github.com/sanger-tol/PretextView
OATK	0.2	https://github.com/c-zhou/oatk
purge_dups	1.2.3	https://github.com/dfguan/purge_dups
samtools	1.16.1, 1.17, and 1.18	https://github.com/samtools/samtools
sanger-tol/genomenote	1.1.1	https://github.com/sanger-tol/genomenote
sanger-tol/readmapping	1.2.1	https://github.com/sanger-tol/readmapping
Seqtk	1.3	https://github.com/lh3/seqtk
Singularity	3.9.0	https://github.com/sylabs/singularity
TreeVal	1.0.0	https://github.com/sanger-tol/treeval
YaHS	1.1a.2	https://github.com/c-zhou/yahs

### Genome annotation

The Ensembl Genebuild annotation system ( Aken *et al.*, 2016) was used to generate annotation for the *Pyrus communis* assembly (GCA_963583255.1) in Ensembl Rapid Release at the EBI. Annotation was created primarily through alignment of transcriptomic data to the genome, with gap filling via protein-to-genome alignments of a select set of proteins from UniProt ( UniProt Consortium, 2019).

### Wellcome Sanger Institute – Legal and Governance

The materials that have contributed to this genome note have been supplied by a Darwin Tree of Life Partner. The submission of materials by a Darwin Tree of Life Partner is subject to the **‘Darwin Tree of Life Project Sampling Code of Practice’**, which can be found in full on the Darwin Tree of Life website here. By agreeing with and signing up to the Sampling Code of Practice, the Darwin Tree of Life Partner agrees they will meet the legal and ethical requirements and standards set out within this document in respect of all samples acquired for, and supplied to, the Darwin Tree of Life Project.

Further, the Wellcome Sanger Institute employs a process whereby due diligence is carried out proportionate to the nature of the materials themselves, and the circumstances under which they have been/are to be collected and provided for use. The purpose of this is to address and mitigate any potential legal and/or ethical implications of receipt and use of the materials as part of the research project, and to ensure that in doing so we align with best practice wherever possible. The overarching areas of consideration are:

•   Ethical review of provenance and sourcing of the material

•   Legality of collection, transfer and use (national and international)

Each transfer of samples is further undertaken according to a Research Collaboration Agreement or Material Transfer Agreement entered into by the Darwin Tree of Life Partner, Genome Research Limited (operating as the Wellcome Sanger Institute), and in some circumstances other Darwin Tree of Life collaborators.

## Data Availability

European Nucleotide Archive: Pyrus communis (pear). Accession number PRJEB65225; https://identifiers.org/ena.embl/PRJEB65225. The genome sequence is released openly for reuse. The *Pyrus communis* genome sequencing initiative is part of the Darwin Tree of Life (DToL) project. All raw sequence data and the assembly have been deposited in INSDC databases. The genome will be annotated using available RNA-Seq data and presented through the Ensembl pipeline at the European Bioinformatics Institute. Raw data and assembly accession identifiers are reported in Table 1.

## Author information

Members of the Royal Botanic Garden Edinburgh Genome Acquisition Lab are listed here: https://doi.org/10.5281/zenodo.4786682.

Members of the Plant Genome Sizing collective are listed here: https://doi.org/10.5281/zenodo.7994306.

Members of the Darwin Tree of Life Barcoding collective are listed here: https://doi.org/10.5281/zenodo.12158331

Members of the Wellcome Sanger Institute Tree of Life Management, Samples and Laboratory team are listed here: https://doi.org/10.5281/zenodo.12162482.

Members of Wellcome Sanger Institute Scientific Operations: Sequencing Operations are listed here: https://doi.org/10.5281/zenodo.12165051.

Members of the Wellcome Sanger Institute Tree of Life Core Informatics team are listed here: https://doi.org/10.5281/zenodo.12160324.

Members of the Tree of Life Core Informatics collective are listed here: https://doi.org/10.5281/zenodo.12205391.

Members of the Darwin Tree of Life Consortium are listed here: https://doi.org/10.5281/zenodo.4783558.
