# Supplementary material for: The genome sequence of the pear, Pyrus communis L
Source: Wellcome Open Res. 2024 Dec 3;9:709. [Version 1] doi: 10.12688/wellcomeopenres.23426.1 (PMC11803386; doi:10.12688/wellcomeopenres.23426.1)
Supplement: Peer review history

# Reviewer report: Reviewer response for version 1

**DOI:** 10.21956/wellcomeopenres.25836.r114444

**Published:** 2025-10-14

**Authors:** Liangsheng Zhang

**Affiliations:** 1 Genomics and Genetic Engineering Laboratory of Ornamental Plants, College of Agriculture and Biotechnology, Zhejiang University, Hangzhou, Zhejiang, China

The genome of *Pyrus communis* generally has a relatively high heterozygosity. It is recommended to retain the two haplotypes generated after hifiasm assembly.

 It is excellent that the article provides genome annotation. It is recommended that a BUSCO assessment be also performed for proteins and compared with the BUSCO results of the genome.

Are sufficient details of methods and materials provided to allow replication by others?

Yes

Is the rationale for creating the dataset(s) clearly described?

Yes

Are the datasets clearly presented in a useable and accessible format?

Yes

Are the protocols appropriate and is the work technically sound?

Yes

Reviewer Expertise:

plant genomes

I confirm that I have read this submission and believe that I have an appropriate level of expertise to confirm that it is of an acceptable scientific standard, however I have significant reservations, as outlined above.

# Reviewer report: Reviewer response for version 1

**DOI:** 10.21956/wellcomeopenres.25836.r114438

**Published:** 2024-12-17

**Authors:** Xin Qiao

**Affiliations:** 1 Nanjing Agricultural University, Nanjing, China

This article reports a new assembly and annotation of European pear based on PacBio HiFi sequencing. I think the quality of genome assembly exceeds previously reported  European pear genomes. The presentation of results and methods is clear and the genome data is publicly available. I have only a minor concern:

 The author should cite previously published three European pear genomes in Background section and add a table to compare assembly metrics among different genomes.

 1.Linsmith, Gareth et al. (2019)

 2.Zhang, Huiting et al. (2022)

 3.Qi, Yongjie et al. (2024)

 To promote genome data dissemination and accessibility in Rosaceae community, the author can submit genome data to GDR database.

Are sufficient details of methods and materials provided to allow replication by others?

Yes

Is the rationale for creating the dataset(s) clearly described?

Yes

Are the datasets clearly presented in a useable and accessible format?

Yes

Are the protocols appropriate and is the work technically sound?

Yes

Reviewer Expertise:

plant comparative genomics, phylogenomics, genome evolution

I confirm that I have read this submission and believe that I have an appropriate level of expertise to confirm that it is of an acceptable scientific standard.

## References

[1] : Pseudo-chromosome-length genome assembly of a double haploid "Bartlett" pear (Pyrus communis L.). *Gigascience* .2019;8(12) : 10.1093/gigascience/giz138 10.1093/gigascience/giz138 31816089 PMC6901071

[2] : Building a foundation for gene family analysis in Rosaceae genomes with a novel workflow: A case study in Pyrus architecture genes. *Front Plant Sci* .2022;13: 10.3389/fpls.2022.975942 975942 10.3389/fpls.2022.975942 36452099 PMC9702816

[3] : Telomere-to-telomere Genome Assembly of two representative Asian and European pear cultivars. *Sci Data* .2024;11(1) : 10.1038/s41597-024-04015-3 1170 10.1038/s41597-024-04015-3 39461942 PMC11513147

# Reviewer report: Reviewer response for version 1

**DOI:** 10.21956/wellcomeopenres.25836.r115245

**Published:** 2024-12-16

**Authors:** Peng Wang

**Affiliations:** 1 Central Public-interest Scientific Institution Basal Research Fund for Chinese Academy of Tropical Agricultural Sciences, Haikou, Hainan, China

The article demonstrates great value for the community of pear science and breeding. As the authors state, there is huge diversity of the cultivated pear, and the origin and evolution of the species is still obscure. The availability of a new genome sequence of the species is valuable to solve these issues. I suggest the authors to cite a paper which has been published earlier on a release of the cultivated pear genome ( https://doi.org/10.1093/g3journal/jkae003).

Are sufficient details of methods and materials provided to allow replication by others?

Yes

Is the rationale for creating the dataset(s) clearly described?

Yes

Are the datasets clearly presented in a useable and accessible format?

Yes

Are the protocols appropriate and is the work technically sound?

Yes

Reviewer Expertise:

Genomics

I confirm that I have read this submission and believe that I have an appropriate level of expertise to confirm that it is of an acceptable scientific standard.

## References

[1] : A chromosome-scale assembly for 'd'Anjou' pear. *G3 (Bethesda)* .2024;14(3) : 10.1093/g3journal/jkae003 10.1093/g3journal/jkae003 38190814 PMC10917493
